# Different prevalence of T2DM risk alleles in Roma population in comparison with the majority Czech population

**DOI:** 10.1002/mgg3.1361

**Published:** 2020-06-24

**Authors:** Jaroslav A. Hubáček, Lenka Šedová, Věra Olišarová, Věra Adámková, Valérie Tóthová

**Affiliations:** ^1^ Centre for Experimental Medicine Institute for Clinical and Experimental Medicine Prague Czech Republic; ^2^ Faculty of Health and Social Sciences University of South Bohemia České Budějovice Czech Republic; ^3^ Department of Preventive Cardiology Institute for Clinical and Experimental Medicine Prague Czech Republic

**Keywords:** Czech population, gene score, polymorphism, Roma population, T2DM

## Abstract

**Background:**

The Czech governmental study suggests up to a 25% higher prevalence of type 2 diabetes mellitus (T2DM) in the Roma population than within the majority population. It is not known whether and to what extent these differences have a genetic background.

**Methods:**

To analyze whether the frequencies of the alleles/genotypes of the *FTO*, *TCF7L2*, *CDKN2A/2B*, *MAEA*, *TLE4*, *IGF2BP2*, *ARAP1,* and *KCNJ11* genes differ between the two major ethnic groups in the Czech Republic, we examined them in DNA samples from 302 Roma individuals and 298 Czech individuals.

**Results:**

Compared to the majority population, Roma are more likely to carry risk alleles in the *FTO* (26% vs. 16% GG homozygotes, *p* < .01), *IGF2BP2* (22% vs. 10% TT homozygotes, *p* < .0001), *ARAP1* (98% vs. 95% of A allele carriers, *p* < .005), and *CDKN2A/2B* (81% vs. 66% of TT homozygotes, *p* < .001) genes; however, less frequently they are carriers of the *TCF7L2* risk allele (34% vs. 48% of the T allele *p* < .0005). Finally, we found significant accumulation of T2DM‐associated alleles between the Roma population in comparison with the majority population (25.4% vs. 15.2% of the carriers of at least 12 risk alleles; *p* < .0001).

**Conclusion:**

The increased prevalence of T2DM in the Roma population may have a background in different frequencies of the risk alleles of genes associated with T2DM development.

## Graphical abstract

The frequency of most T2DM‐associated genotypes differed significantly between the Czech majority population and Roma. Roma subjects have in mean increased numbers of risky alleles. Likely, genes are in background of the increased T2DM incidence in this ethnic group.

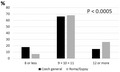

## 1 INTRODUCTION

Type 2 diabetes mellitus (T2DM, previously known as insulin‐independent diabetes) is a common civilization disease that affects the metabolism of glucose. T2DM is characterized by insulin deficiency, which is caused by pancreatic β‐cell dysfunction and insulin resistance in organs. T2DM patients have increased blood glucose concentrations, and they suffer from many microvascular complications, such as diabetic nephropathy, neuropathy, and retinopathy (Zheng, Ley, & Hu, 2018). Diabetes is one of the major risk factors for cardiovascular diseases; among patients with myocardial infarction or stroke, there is a significantly higher prevalence of T2DM in comparison to the general population (Bhupathiraju & Hu, 2016; Hubacek et al., 2017; Peters, Huxley, & Woodward, 2014).

The incidence of T2DM in industrial countries is estimated to be approximately 8% in Caucasians (Emerging Risk Factors Collaboration, 2010), but it significantly differs between different ethnic groups. The highest prevalence has been described in populations surrounding the Persian Gulf, reaching almost 30% (Alhyas, McKay, & Majeed, 2012). Our awareness of the prevalence of T2DM in the Roma minorities is sparse. In fact, there are just a few studies focused on this topic (reviewed by Nunes, Kučerová, Lukáč, Kvapil, & Brož, 2018; Vozarova de Courten et al., 2003); unfortunately, they do not reach the representative sample standards and are low in number. Nevertheless, they suggest that the prevalence of diabetes in Roma increased in comparison with the general majority populations. In agreement with these observations, in the Czech Republic, the governmental study suggests up to a 25% higher prevalence of T2DM in the Roma minority than in the majority population (http://www.mzcr.cz/verejne/dokumenty/zprava‐o‐zdravi‐obyvatel‐ceske‐republiky2014‐_9420_3016_5.html [document in Czech, accessed November 2019]).

The pathogenesis of T2DM development is multifactorial with both genetic and environmental (agricultural policies, physical activity, sleep, food availability, and environment) factors (Bhupathiraju & Hu, 2016). It is not known whether and to what extent the interethnic differences in T2DM prevalence reflect the different genetic backgrounds between the examined populations and to what extent unhealthy lifestyle could be responsible.

Genome‐wide association studies detected dozens of single nucleotide polymorphisms (SNPs) within the different genes that are associated with an increased risk of T2DM (Kodama et al., 2018; McCarthy, 2017). Talmud et al. (2015) summarized a list of 65 SNPs and created a gene score that could be informative for the estimation of increased T2DM risk.

Based on the genome‐wide association studies (GWAS) results and especially on the published T2DM‐associated gene score (Kodama et al., 2018; McCarthy, 2017; Talmud et al., 2015), we selected SNPs within the genes for *FTO* (OMIM acc. No. 610966; nucleic acid demethylase; rs17817449), TCF7L2 (OMIM acc. No. 602228: transcription factor implicated in blood glucose homeostasis; rs7903146), CDKN2A/2B (OMIM acc. No. 60060 and 600431; cyclin‐dependent kinase inhibitors; rs10811661), MAEA (OMIM acc. No: 606801; erythroblast macrophage protein, homologue of the yeast glucose‐induced degradation‐deficient protein; rs6819243), TLE4 (OMIM acc. No: 605132; complex locus of several transcription factors; rs17791513), IGF2BP2 (OMIM acc. No: 608289; insulin‐like growth factor 2 mRNA‐binding protein 2; rs4402960), ARAP1 (alias CENTAURIN, OMIM acc. No: 606646; possibly involved in cell signaling and communication; rs1552224), and KCNJ11 (OMIM acc. No: 600358; kidney potassium channel; rs5215), which are considered to be the most powerful genetic determinants of T2DM development in Caucasians.

Our study was focused on the analysis of the differences in the frequencies of genotypes of the *FTO*, *TCF7L2*, *CDKN2A/2B*, *MAEA*, *TLE4*, *IGF2BP2*, *ARAP1,* and *KCNJ11* genes between two major ethnic groups living in the Czech Republic. We tested the theory that there are significant differences in genotype frequencies of these genes between the Roma minority inhabiting the region of the Czech Republic and the majority of the Czech population.

## 2 MATERIAL AND METHODS

### 2.1 Ethical compliance

All subjects involved in the study provided written informed consent. The study protocol was approved by the institutional ethics committee and conducted according to the Good Clinical Practice guidelines and in agreement with the Helsinki Declaration of 1975.

### 2.2 Study populations

We examined two ethnically distinct populations inhabiting an identical region of South Bohemia (Table 1). Six hundred unrelated adults (at least 18 years old at the time of examination) were included in the study (Adámková et al., 2015; Hubacek et al., 2018; Šedová et al., 2015). Czech Roma (*N* = 302) were recruited using snowball sampling (Hughes, Fenton, Hine, Pilgrim, & Tibbs, 1995) and Czech Caucasians/Slavs (*N* = 298) were recruited using quota sampling (for more details, see Adámková et al., 2015; Šedová et al., 2015; Walter, 1989). Ethnicity was based on self‐reported information.

**Table 1:** General characteristics of the examined subjects

	Roma	Majority slavs
*N*	302	298
Age (y)	39.2 ± 12.8	39.5 ± 15.1
% of females	50	50
BMI (kg/m^2^)	29,9 ± 5,6	25.0 ± 6,0
SBP (mm Hg)	124,3 ± 20,9	124.9 ± 14.4
DBP (mm Hg)	76,6 ± 12,71	77.2 ± 10.6
Total cholesterol (mmol/L)	5.1 ± 1.4	5.1 ± 1.1
Glycemia 6 mmol/L and more (%)	58.6	23.3
Total body fat 25% and more (%)	69.4	61.7
% of alcohol consumers^a^	10.5	16.4

^a^ Defined as a consumption the day before the examination

### 2.3 DNA analyses

DNA was isolated using the “Xtreme DNA Isolation Kit” from buccal cells obtained through “DNA buccal swabs” (both Isohelix, Cell Projects Ltd, UK) according to conditions specified by the manufacturer.

Genotypes of interest were analyzed using the polymerase chain reaction‐restriction fragment length polymorphism (PCR‐RFLP) method (polymorphisms within the *FTO* (Hubáček et al., 2015), *TCF7L2*, *CDKN2A/2B*, *MAEA*, *IGF2BP2,* and *TLE4* or by TaqMan assays (*KCNJ11* and *ARAP1* variants). Restriction fragments were separated using a 10% polyacrylamide gel. Fermentas International Inc. (Burlington, Ontario, Canada) provided all PCR chemicals, and PCRs were performed on the MJ Research DYAD Disciple PCR device.

Details about the oligonucleotides and restriction enzymes were used, PCR conditions or TaqMan assay ID numbers are summarized in Table 2.

**Table 2:** Genotyping details for analysis of SNPs of interest

Polymorphism	Primer sequences or Taqman assay ID	PCR product	Enzyme	Size of restriction fragments (bp)	Allele	Effect size per RA^9−11,30^
*FTO* rs17817449	5′ ggtgaagaggaggagattgtgtaactgg 5′ gaagccctgagaagtttagagtaaattggg	198 bp	AlwNI	198 99 + 99	**G** T	~1.2–1.5
*TCF7L2* rs7903146	5′ gaacaattagagagctaagcactttttaggta 5′ tgtccagggcccctctaacctt	155 bp	Rasi	155 123 + 32	**T** C	~1.3–2.0
*CDKN2A/2B* rs10811661	5′ tgaagacattagaacaccataacctttcc 5′ taggaggagccagaagacagatcagg	143 bp	BspHI	143 94 + 49	C **T**	~ 1.2–1.5
*MAEA* rs6819243	5′ aaccttcaggttggccttgatgcctc 5′ ttcatccccaggtcaggaaactcc	259 bp	MvaI	189 + 70 95 + 94 + 70	**T** C	~1.2
*IGF2BP2* rs4402960	5′ aagcaggtttggaaccctggaattcc 5′ tctagcctgggcgacagagcaagactcc	220 bp	MseI	220 111 + 109	G **T**	~1.2
*TLE4* rs17791513	5′ aagagagtgctgaataggaaccatttgtcc 5′ taatgcttcagactatatccccttatcga	136 bp	Bsu15I	136 107 + 29	G **A**	~1.2–1.5
*KCNJ11* rs5215	C_2991148_10	n.a.	n.a.	n.a.	**C** T	~1.15
*ARAP1* rs1552224	C_1953903_10	n.a.	n.a.	n.a.	**A** C	~1.15

Note

T2DM‐associated allele is in bold. RA – risky allele; rs17817449 – NC_000016.10:g.53779455T>G; rs7903146 – NC_000010.11:g.112998590C>T; rs10811661 – NC_000009.12:g.22134095T>C; rs6819243 – NC_000004.12:g.1299457T>C; rs4402960 – NC_000003.12:g.185793899G>T, rs17791513 – NC_000009.12:g.79290675A>G; rs5215 – NC_000011.10:g.17387083C>T; rs1552224 – NC_000011.10:g.72722053A>C.

### 2.4 Statistical analysis

The deviance of genotype frequencies among the groups was analyzed according to Hardy‐Weinberg equilibrium (www.tufts.edu/~mcourt01/Documents/Court%20lab%20‐%20HW%20calculator.xls). Differences in allelic and genotype frequencies were compared using an online chi‐square test (www.socsistatistics.com). Comparisons were performed in “AA vs. Aa vs. aa” model. In the cases where fewer than five subjects were present in some genotype category in at least one group, the rare homozygotes were pooled with heterozygotes. To calculate the unweighted gene score, the number of risk alleles [risk category was based on the publication of Talmud et al. (2015)] was summarized for each subject, and the values were compared between the ethnicities using a chi‐square test. For this comparison, subjects with more than one missing genotype were excluded, leaving 287 subjects (96.3%) in the non‐Roma majority Slavs and 295 (97.7%) subjects in the Roma minority population. The missing genotypes were artificially imputed as described in detail in Hubacek et al. (2019) Altogether, 48 genotyping results (1.05%) were imputed, 26 in the majority (1.13%), and 22 in Roma subjects (0.93%). In the majority, most of the imputations (*N* = 12) were necessary in the *CDKN2A/2B* gene (rs10811661), and in Roma subjects, 11 imputations were necessary for the *TCF7L2* (rs7903146) gene.

As there were very low numbers of subjects with 6 and 7 risk alleles, they comprised, together with carriers of 8 risk alleles, the “low‐risk” allele subgroup. In contrast, subjects with 12–16 alleles comprised the “high‐risk” allele subgroup. The mean and S.D. of risk alleles were calculated for each ethnic group and compared by the two‐tailed *t*‐test.

Due to the large number of comparisons, a *p* < 0.01 was considered significant.

### 2.5 Data availability

The data that support the findings of this study are available on request from the corresponding author at jahb@kem.cz. The data are not publicly available due to privacy and ethical reason.

## 3 RESULTS

The achieved call rates for individual SNPs were between 92.9% and 100% in the Czech majority population and between 93.7% and 99.4% in the Roma population. Within the individual ethnic groups, no significant gender differences in genotype frequencies were observed (results not presented in detail).

### 3.1 Individual SNPS

The frequency of genotypes of most analyzed variants differed significantly between the examined ethnicities (for more details see Table 3). Compared to the majority population, Roma were more likely to carry risk alleles/genotypes of the *FTO* (26% vs. 16% GG homozygotes, *p* < .01), *CDKN2A/2B* (81% vs. 66% TT homozygotes, *p* < .001), *IGF2BP2* (22% vs. 10% TT homozygotes, *p* < .0001), and *ARAP1* (98% vs. 95% A allele carriers, *p* < .005) genes.

**Table 3:** Genotype frequencies of examined polymorphisms within the majority Czech general population and Roma population

	Czech majority				Roma minority					*p*
	*N*	%	CR	HWE	*N*	%	CR		HWE	
*FTO*										
rs17817449										
**GG**	47	15.8			76	25.3				
**G**T	161	54.0	100.0	0.08	154	51.3	99.4		0.64	.009
TT	90	30.2			70	23.3				
*TCF7L2*										
rs7903146										
CC	151	51.9			186	65.7				
C**T**	120	41.3	97.7	0.56	93	32.9	93.7		0.05	.0008
**TT**	20	6.9			4	1.4				
*CDKN2A/2B*										
rs10811661										
**TT**	188	67.9			235	81.3				
**T**C	79	28.5	92.9	0.64	51	17.6	95.7		0.90	.0002
CC	10	3.6			3	1.0				
*MAEA*										
rs6819243										
**TT**	275	94.5			273	92.5				
**T**C	16	5.5	97.7	0.63	22	7.5		97.7	0.51	.34
CC	0	0.0			0	0.0				
*TLE4*										
rs17791513										
**AA**	260	88.7			271	90.9				
**A**G	32	10.9	98.3	0.99	26	8.7		98.7	0.67	.38
GG	1	0.4			1	0.3				
*KCNJ11*										
rs5215										
**CC**	113	39.8			121	40.5				
**C**T	121	42.6	95.3	0.08	131	43.8		99.0	0.25	.83
TT	50	17.6			47	15.7				
*IGF2BP2*										
rs4402960										
GG	134	46.4			84	28.2				
G**T**	125	43.3	97.0	0.91	149	50.0		98.7	0.94	.0001
**TT**	30	10.4			65	21.8				
*ARAP1*										
rs1552224										
**AA**	204	70.1			235	80.2				
**A**C	72	24.7	97.7	0.01	53	18.1		97.0	0.33	.007
CC	15	5.2			5	1.7				

Note

T2DM‐associated allele is in bold. rs17817449 – NC_000016.10:g.53779455T>G; rs7903146 – NC_000010.11:g.112998590C>T; rs10811661 – NC_000009.12:g.22134095T>C; rs6819243 – NC_000004.12:g.1299457T>C; rs4402960 – NC_000003.12:g.185793899G>T, rs17791513 – NC_000009.12:g.79290675A>G; rs5215 – NC_000011.10:g.17387083C>T; rs1552224 – NC_000011.10:g.72722053A>C.

Abbreviations: CR – call rate; HWE – Hardy–Weinberg equilibrium.

In contrast, in the Roma population, there were fewer carriers of the *TCF7L2* risk allele (34% vs. 48% of the T allele *p* < .0005) in comparison with the majority population.

Finally, we did not detect differences between the Czech majority population and Roma living in the Czech Republic for the allelic/genotype frequencies of the polymorphisms within the *MAEA*, *TLE4,* and *KCNJ11* genes. For the first two mentioned, the cause could be the generally low frequency (in both cases below 6% in both ethnicities) of the minor allele, suggesting the possible insufficient power of the study.

### 3.2 Gene score

**Figure 1:**
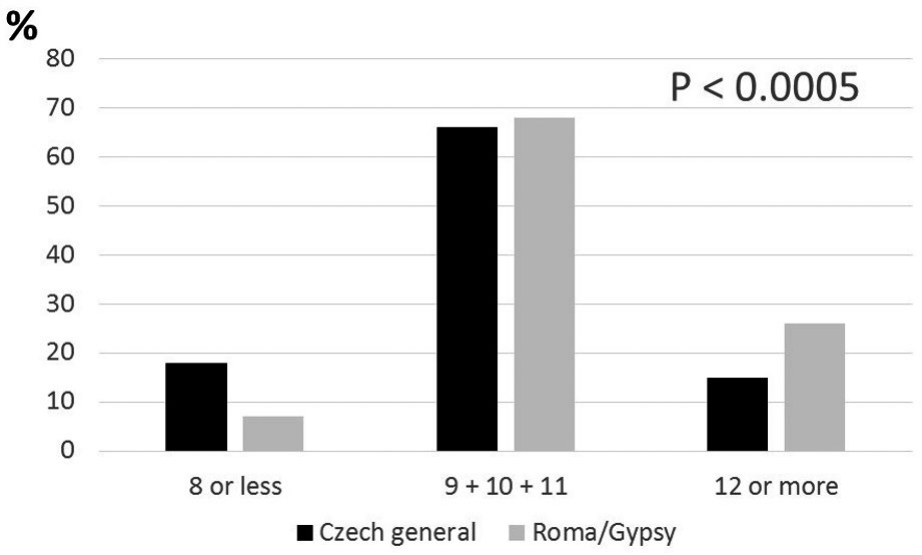
Differences in unweighted T2DM gene score between the Roma minority and non‐Roma majority inhabiting the identical middle European region

The cumulative prevalence of the risk alleles within both examined groups is summarized in Table 4 and Figure 1. The span of the numbers of risk alleles was wide. The minimum observed was, however, relatively high. Everybody was a carrier of at least 6 risk alleles, and one subject was carrier of all possible 16 risk alleles. The unweighted gene score values were between 6 and 16 in the Czech majority population and between 7 and 15 in the Roma population. Means of risk alleles differed significantly (*p* < .00005) between the two groups. There were 9.97 ± 2.28 risk alleles per person in the majority and 10.52 ± 2.25 risk alleles per person within the Roma subjects.

**Table 4:** T2DM‐unweighted gene score in the Roma population and the majority population

*N*	6		7		8		9		10		11		12		13		14		15		16	
	*N*	%	*N*	%	*N*	%	*N*	%	*N*	%	*N*	%	*N*	%	*N*	%	*N*	%	*N*	%	*N*	%
Majority	8	2.8	11	3.8	34	11.8	53	18.5	69	24.0	68	23.7	30	10.5	9	3.1	3	1.0	1	0.3	1	0.3
Minority	0	0.0	7	2.4	13	4.4	52	17.6	83	28.1	65	22.0	49	16.6	16	5.4	9	3.1	1	0.3	0	0.0

Abbreviation: *N*, number of risk alleles.

There was a significant difference (*p* < .0001) in the unweighted gene score value distribution between the Czech majority population and the Roma population in predefined subgroups (Figure 1).

Within the low‐risk group range (8 risk alleles maximum), there were 18.4% of majority subjects and 6.6% of Roma subjects only. In contrast, within the high‐risk group category (at least 12 risk alleles), 15.2% were the majority, and 25.4% were Roma subjects.

## 4 DISCUSSION

The results of our study support the theory that the increased prevalence of T2DM in the Roma population may be associated with different frequencies of the risk alleles of some genes associated with T2DM development.

Our study is in contrast with a recently published study focused on similar topics in the Hungarian majority and Roma populations (Werissa et al., 2019). They found slightly increased accumulation of T2DM‐associated alleles within the majority population. This discrepancy with our study, however, could be based simply on the selection of genes/variants. The Hungarian authors selected 16 SNPs, but only two of them (within the *TCF7L2* and *CDKN2A/2B* genes) were identical to our study. Interestingly, the frequencies of SNPs within these genes differ to some extent in our study and the Hungarian study. For the general Czech and Hungarian population, the minor *TCF7L2* allele frequencies are highly similar (27.5% vs. 29.5%), but Hungarian Roma have a higher prevalence of the risk allele (24.2% vs. 17.8%) than Roma inhabiting the Czech region. For *CDK2A/2B*, the majority populations had frequencies of the risk allele of 82.8% and 82.1%, and Czech Roma were carriers of this allele (90.1%) slightly more often than Hungarian Roma (85.3). Even these incomplete comparisons suggest that there could be increased genetic heterogeneity between different Roma subpopulations across Europe. Another important issue that needs to be mentioned is the fact that a previous study (Nagy et al., 2011) suggests the Asian origin of Hungarians; thus, the genetic background of different ethnicities in the Hungarian geographic region could be historically more similar than that in other European regions.

Of the analyzed SNPs/genes, *FTO* is probably the most commonly studied. Minor alleles are associated with an increased risk of T2DM as well as increased risk of T2DM complications (Gaulton, 2017; Hubacek et al., 2018). *FTO* variants are also discussed as potential predictors of obesity treatment (Xiang et al., 2016; Zlatohlavek et al., 2013). *FTO* polymorphisms are the only SNPs whose frequencies were analyzed previously in more Roma populations from different regions. The prevalence of minor allele homozygotes of the rs9939609 variant (which is in non‐Roma Caucasians in almost complete LD with rs17817449 variant) was 22% in the Slovak‐Roma population (Mačeková et al., 2012) and, as expected, significantly associated with increased BMI values. A similar association between increased BMI and the *FTO* rs9939609 variant was observed in the Hungarian Roma (Nagy, Fiatal, Sándor, & Ádány, 2017) (where the prevalence of the *FTO* risk allele is higher) but not in the Spanish Roma population (Poveda, Ibáñez, & Rebato, 2014). These results suggest a relatively high *FTO* polymorphism heterogeneity effect between the Roma populations (or possibly, not such strong LD between rs9939609 and rs17817449 polymorphisms) in comparison with the European majority populations.

The *TCF7L2* gene seems to be the strongest genetic predictor of T2DM development (Adams & Vella, 2018). T2DM‐associated alleles of this gene are associated with reduced β‐cell function (reviewed by Adams & Vella, 2018). Most interestingly, in this study, we found a higher frequency of the C allele within the Roma population. This allele is described as protective against T2DM development, and this had been confirmed also in the Czech population (Včelák et al., 2012).

The importance of *CDKN2A/2B* in T2DM pathology is not clear, albeit rs10811661 was identified in the first GWAS presented among the most powerful signals (Cauchi et al., 2008). However, there seems not to be an association between this variant and an increased risk of T2DM in the Czech population (Hubáček, Neškudla, Klementová, Adámková, & Pelikánová, 2013) and similarly controversial results have also been published for the Arab population (Nemr et al., 2012). In contrast, an association between this polymorphism and T2DM was found in an Asian Indian (Chidambaram et al., 2016) and a Pakistani populations (Rees et al., 2011), which are populations from the geographical region where the European Roma seem to originate.

Most of the studies of *IGF2BP2* are focused on gestational diabetes, but a global meta‐analysis confers an association with T2DM both for Caucasians and for Indian populations (Zhao et al., 2012).

For the *ARAP1* (alias *CENTD2*) variant, the vast majority (>95%) of subjects in both ethnicities are carriers of at least one risk allele here. This makes the utility of this variant questionable for T2DM estimation at the population level. The major allele is associated with reduced insulin release (Nielsen et al., 2011) and was associated with protection from T2DM in northern‐Indian women using the WHO 2013 (but not using the WHO 1999) criteria (Arora et al., 2018).

The T2DM‐associated risk potential of only four genes of our set [for *FTO* (Hubacek et al., 2018), *IGF2BP2*, (Gu et al., 2012), *CDKN2A/2B* (Hubáček et al., 2013), and *TCF7L2* (Včelák et al., 2012)] was examined in the Czech majority population. Thus, although we are aware of the potential inaccuracy, for creation of the unweighted gene score, we have used the risk status based on the study of Talmud et al. (2015). The use of aggregated risk score, summarizing the effects of more genes in one single value, should improve the predictive ability of genetic testing. Here, we clearly show that Roma subjects are carriers of more T2DM‐associated alleles.

Our study is not the only one that has described the differences in genetic background between the Roma and majority Caucasian populations.

For example, in Roma, allelic differences in lactate dehydrogenase (Hubácek et al., 2017b), drug metabolism enzymes (Tomas et al., 2017, Dlouhá et al., in press), or osteoprotegerin (Mydlárová Blaščáková et al., 2017) polymorphisms clearly underlie genetic differences that could have important clinical (optimal treatment or dietary recommendations) or live expectancy consequences.

Previous studies and our results show that the Roma are genetically distinct from both the European majority population and the original Asian populations, where the origin of European Roma gypsies is dated. The differences could be due to random genetic drift, mostly the founder effects rather than unlikely selection pressure or environment adaptation, as these factors were identical in past centuries for majority and minority populations inhabiting the same European region.

We are aware of the limitations of our study. The major one is that for the gene score calculation, we had to use the results obtained for other European populations. Nevertheless, it cannot be excluded that (especially in the case of the Roma minority) the associations between the analyzed genes and T2DM could differ from the expected directions. We have not confirmed the association with T2DM for the *CDKN2A/2B* rs10811661 SNP (Hubáček et al., 2013). The status between the risk of T2DM in the Czech population is not known for the rest of the analyzed genes; thus, the potential limitation of the present study is that we used allelic gene score based on results obtained from other population (Talmud et al., 2015) and we decide not to use the weighted gene score which takes into account also the effect size of individual gene variants. This, however, does not reduce the importance of our findings; in fact, quite the contrary. If in the future the genetic risk score will be used for risk estimation, the analysis needs to be performed in young asymptomatic subjects, where the weighted gene values would be definitely different from the values obtained for the population, where increased nongenetic risk factors are presented.

The genetic tools, likely the population‐specific genetic risk scores, in the future could improve the in‐time identification of the high‐risk subsets of the population. Accumulating evidence suggests that genetic testing will have an irreplaceable role in personalized therapy and clinical decision making (Rodríguez Vicente, Herrero Cervera, Bernal, Rojas, & Peiró, 2018). To achieve this goal, examinations of a higher number of genetic polymorphisms, especially in minorities, interaction analysis, and detailed population‐specific gene score calculation are the further necessary steps. It is clear that there are important differences between populations and ethnicities.

We conclude that there is a significant genetic difference between the Czech majority population and the Roma population. The increased prevalence of T2DM in the Roma population could be based on an increased cumulative number of T2DM‐associated common alleles within different genes.

## CONFLICT OF INTEREST

The authors declare no conflict of interest.

## AUTHOR CONTRIBUTION

JAH – Writing – Original Draft; Methodology; Investigation – genotyping; Funding acquisition; Supervision; Data analysis; Conceptualization. LŠ – Writing – Review & Editing; Investigation; Data Curation. VO – Writing – Review & Editing; Investigation; Data Curation. VA – Writing – Review & Editing; Methodology; Investigation; Resources; Supervision; Conceptualization. VT – Writing – Review & Editing; Funding acquisition; Methodology; Investigation; Resources; Project administration; Visualization; Formal analysis; Conceptualization.

## References

[mgg31361-bib-0001] Adámková, V. , Hubáček, J. A. , Nováková, D. , Dolák, F. , Adámek, V. , Lánská, V. , … Šedová, L. (2015). Genetic and biochemical characteristics in the Roma minority in the South Bohemia Region. Neuro Endocrinology Letters, 36(Suppl 2), 29–34. 26748524

[mgg31361-bib-0002] Adams, J. D. , & Vella, A. (2018). What can diabetes‐associated genetic variation in TCF7L2 teach us about the pathogenesis of type 2 diabetes? Metabolic Syndrome Related Disorders, 16(8), 383–389. 10.1089/met.2018.0024 29993315 PMC6167616

[mgg31361-bib-0003] Alhyas, L. , McKay, A. , & Majeed, A. (2012). Prevalence of type 2 diabetes in the States of the co‐operation council for the Arab States of the Gulf: A systematic review. PLoS One, 7(8), e40948 10.1371/journal.pone.0040948 22905094 PMC3414510

[mgg31361-bib-0004] Arora, G. P. , Almgren, P. , Brøns, C. , Thaman, R. G. , Vaag, A. A. , Groop, L. , & Prasad, R. B. (2018). Association between genetic risk variants and glucose intolerance during pregnancy in north Indian women. BMC Medical Genomics, 11(1), 64 10.1186/s12920-018-0380-8 30089489 PMC6083526

[mgg31361-bib-0005] Bhupathiraju, S. N. , & Hu, F. B. (2016). Epidemiology of obesity and diabetes and their cardiovascular complications. Circulation Research, 118(11), 1723–1735. 10.1161/CIRCRESAHA.115.306825 27230638 PMC4887150

[mgg31361-bib-0006] Cauchi, S. , Meyre, D. , Durand, E. , Proença, C. , Marre, M. , Hadjadj, S. , … Froguel, P. (2008). Post genome‐wide association studies of novel genes associated with type 2 diabetes show gene‐gene interaction and high predictive value. PLoS One, 3(5), e2031 10.1371/journal.pone.0002031 18461161 PMC2346547

[mgg31361-bib-0007] Chidambaram, M. , Liju, S. , Saboo, B. , Sathyavani, K. , Viswanathan, V. , Pankratz, N. , … Radha, V. (2016). Replication of genome‐wide association signals in Asian Indians with early‐onset type 2 diabetes. Acta Diabetologica, 53(6), 915–923. 10.1007/s00592-016-0889-2 27488727

[mgg31361-bib-0008] Dlouhá, L. , Adámková, V. , Šedová, L. , Olišarová, V. , Hubáček, J. A. , & Tóthová, V. Five genetic polymorphisms of cytochrome P450 enzymes in the Czech non‐Roma and Czech Roma population samples. Drug Metabolism and Personalized Therapy, In press. 10.1515/dmpt-2020-0103 32681777

[mgg31361-bib-0009] Emerging Risk Factors Collaboration . (2010). Diabetes mellitus, fasting blood glucose concentration, and risk of vascular disease: A collaborative meta‐analysis of 102 prospective studies. Lancet (London, England), 375(9733), 2215–2222. 10.1016/S0140-6736(10)60484-9 PMC2904878 20609967

[mgg31361-bib-0010] Gaulton, K. J. (2017). Mechanisms of type 2 diabetes risk loci. Current Diabetes Reports, 17(9), 72 10.1007/s11892-017-0908-x 28741265

[mgg31361-bib-0011] Gu, T. , Horová, E. , Möllsten, A. , Seman, N. A. , Falhammar, H. , Prázný, M. , … Gu, H. F. (2012). IGF2BP2 and IGF2 genetic effects in diabetes and diabetic nephropathy. Journal of Diabetes and Its Complications, 26(5), 393–398. 10.1016/j.jdiacomp.2012.05.012 22770937

[mgg31361-bib-0012] Hubácek, J. A. , Adámková, V. , Šedová, L. , Olišarová, V. , Adámek, V. , & Tóthová, V. (2017b). Frequency of adult type‐associated lactase persistence LCT‐13910C/T genotypes in the Czech/Slav and Czech Roma/Gypsy populations. Genetics and Molecular Biology, 40(2), 450–452. 10.1590/1678-4685-GMB-2016-0071 28497837 PMC5488455

[mgg31361-bib-0013] Hubacek, J. A. , Dlouha, D. , Adamkova, V. , Schwarzova, L. , Lanska, V. , Ceska, R. , … Vrablik, M. (2019). The gene score for predicting hypertriglyceridemia: New insights from a Czech case‐control study. Molecular Diagnosis & Therapy, 23(4), 555–562. 10.1007/s40291-019-00412-2 31222479

[mgg31361-bib-0014] Hubacek, J. A. , Dlouha, D. , Klementova, M. , Lanska, V. , Neskudla, T. , & Pelikanova, T. (2018). The FTO variant is associated with chronic complications of diabetes mellitus in Czech population. Gene, 642, 220–224. 10.1016/j.gene.2017.11.040 29154870

[mgg31361-bib-0015] Hubáček, J. A. , Neškudla, T. , Klementová, M. , Adámková, V. , & Pelikánová, T. (2013). Tagging rs10811661 variant at CDKN2A/2B locus is not associated with type 2 diabetes mellitus in Czech population. Folia Biologica, 59(4), 168–171. 24093775 10.14712/fb2013059040168

[mgg31361-bib-0016] Hubáček, J. A. , Pikhart, H. , Peasey, A. , Kubínová, R. , & Bobák, M. (2015). Nobody is perfect: Comparison of the accuracy of PCR‐RFLP and KASP™ method for genotyping. ADH1B and FTO polymorphisms as examples. Folia Biologica, 61(4), 156–160. 26441205 10.14712/fb2015061040156

[mgg31361-bib-0017] Hubacek, J. A. , Stanek, V. , Gebauerova, M. , Adamkova, V. , Lesauskaite, V. , Zaliaduonyte‐peksiene, D. , … Pitha, J. (2017). Traditional risk factors of acute coronary syndrome in four different male populations ‐ total cholesterol value does not seem to be relevant risk factor. Physiological Research, 66(Suppl 1), S121–S128. 10.33549/physiolres.933597 28379037

[mgg31361-bib-0018] Hughes, A. O. , Fenton, S. , Hine, C. E. , Pilgrim, S. , & Tibbs, N. (1995). Strategies for sampling black and ethnic minority populations. Journal of Public Health Medicine, 17(2), 187–192. 10.1093/oxfordjournals.pubmed.a043091 7576802

[mgg31361-bib-0019] Kodama, S. , Fujihara, K. , Ishiguro, H. , Horikawa, C. , Ohara, N. , Yachi, Y. , … Sone, H. (2018). Quantitative relationship between cumulative risk alleles based on genome‐wide association studies and type 2 diabetes mellitus: A systematic review and meta‐analysis. Journal of Epidemiology, 28(1), 3–18. 10.2188/jea.JE20160151 29093303 PMC5742374

[mgg31361-bib-0020] Mačeková, S. , Bernasovský, I. , Gabriková, D. , Bôžiková, A. , Bernasovská, J. , Boroňová, I. , … Čarnogurská, J. (2012). Association of the FTO rs9939609 polymorphism with obesity in Roma/Gypsy population. American Journal of Physical Anthropology, 147(1), 30–34. 10.1002/ajpa.21604 21989907

[mgg31361-bib-0021] McCarthy, M. I. (2017). Genetics of T2DM in 2016: Biological and translational insights from T2DM genetics. Nature Reviews Endocrinology, 13(2), 71–72. 10.1038/nrendo.2016.212 28051116

[mgg31361-bib-0022] Mydlárová Blaščáková, M. , Blaščáková, Ľ. , Poráčová, J. , Mydlár, J. , Vašková, J. , Bernasovská, J. , … Bernasovský, I. (2017). Relationship between A163G osteoprotegerin gene polymorphism and other osteoporosis parameters in Roma and non‐Roma postmenopausal women in eastern Slovakia. Journal of Clinical Laboratory Analysis, 31(5), e22093 10.1002/jcla.22093 PMC6816896 27859736

[mgg31361-bib-0023] Nagy, D. , Tömöry, G. , Csányi, B. , Bogácsi‐Szabó, E. , Czibula, Á. , Priskin, K. , … Raskó, I. (2011). Comparison of lactase persistence polymorphism in ancient and present‐day Hungarian populations. American Journal of Physical Anthropology, 145(2), 262–269. 10.1002/ajpa.21490 21365615

[mgg31361-bib-0024] Nagy, K. , Fiatal, S. , Sándor, J. , & Ádány, R. (2017). Distinct penetrance of obesity‐associated susceptibility alleles in the Hungarian general and Roma populations. Obesity Facts, 10(5), 444–457. 10.1159/000478094 28988247 PMC5741168

[mgg31361-bib-0025] Nemr, R. , Almawi, A. W. , Echtay, A. , Sater, M. S. , Daher, H. S. , & Almawi, W. Y. (2012). Replication study of common variants in CDKAL1 and CDKN2A/2B genes associated with type 2 diabetes in Lebanese Arab population. Diabetes Research and Clinical Practice, 95(2), e37–e40. 10.1016/j.diabres.2011.11.002 22119613

[mgg31361-bib-0026] Nielsen, T. , Sparsø, T. , Grarup, N. , Jørgensen, T. , Pisinger, C. , Witte, D. R. , … Pedersen, O. (2011). Type 2 diabetes risk allele near CENTD2 is associated with decreased glucose‐stimulated insulin release. Diabetologia, 54(5), 1052–1056. 10.1007/s00125-011-2054-3 21267535

[mgg31361-bib-0027] Nunes, M. A. , Kučerová, K. , Lukáč, O. , Kvapil, M. , & Brož, J. (2018). Prevalence of diabetes mellitus among Roma populations ‐ a systematic review. International Journal of Environmental Research and Public Health, 15(11), 2607 10.3390/ijerph15112607 PMC6265881 30469436

[mgg31361-bib-0028] Peters, S. A. , Huxley, R. R. , & Woodward, M. (2014). Diabetes as risk factor for incident coronary heart disease in women compared with men: A systematic review and meta‐analysis of 64 cohorts including 858,507 individuals and 28,203 coronary events. Diabetologia, 57(8), 1542–1551. 10.1007/s00125-014-3260-6 24859435

[mgg31361-bib-0029] Poveda, A. , Ibáñez, M. E. , & Rebato, E. (2014). Common variants in BDNF, FAIM2, FTO, MC4R, NEGR1, and SH2B1 show association with obesity‐related variables in Spanish Roma population. American Journal of Human Biology, 26(5), 660–669. 10.1002/ajhb.22576 24948161

[mgg31361-bib-0030] Rees, S. D. , Hydrie, M. Z. I. , Shera, A. S. , Kumar, S. , O’Hare, J. P. , Barnett, A. H. , … Kelly, M. A. (2011). Replication of 13 genome‐wide association (GWA)‐validated risk variants for type 2 diabetes in Pakistani populations. Diabetologia, 54(6), 1368–1374. 10.1007/s00125-011-2063-2 21350842

[mgg31361-bib-0031] Rodríguez Vicente, A. E. , Herrero Cervera, M. J. , Bernal, M. L. , Rojas, L. , & Peiró, A. M. (2018). Personalized medicine into health national services: Barriers and potentialities. Drug Metabolism and Personalized Therapy, 33(4), 159–163. 10.1515/dmpt-2018-0017 30391933

[mgg31361-bib-0032] Šedová, L. , Tóthová, V. , Olišarová, V. , Adámkova, V. , Bártlová, S. , Dolák, F. , … Prokešová, R. (2015). Evaluation of selected indicators of overweight and obesity of Roma minority in the region of South Bohemia. Neuro Endocrinology Letters, 36(Suppl 2), 35–42. 26748525

[mgg31361-bib-0033] Talmud, P. J. , Cooper, J. A. , Morris, R. W. , Dudbridge, F. , Shah, T. , Engmann, J. , …, Humphries, S. E. (2015). Sixty‐five common genetic variants and prediction of type 2 diabetes. Diabetes, 64(5), 1830–1840. 10.2337/db14-1504 25475436 PMC4407866

[mgg31361-bib-0034] Tomas, Ž. , Kuhanec, A. , Škarić‐Jurić, T. , Petranović, M. Z. , Narančić, N. S. , Janićijević, B. , & Salihović, M. P. (2017). Distinctiveness of the Roma population within CYP2B6 worldwide variation. Pharmacogenomics, 18(17), 1575–1587. 10.2217/pgs-2017-0105 29095103

[mgg31361-bib-0035] Včelák, J. , Vejražková, D. , Vaňková, M. , Lukášová, P. , Bradnová, O. , Hálková, T. , … Bendlová, B. (2012). T2D risk haplotypes of the TCF7L2 gene in the Czech population sample: The association with free fatty acids composition. Physiological Research, 61(3), 229–240. 10.33549/physiolres.932272 22480428

[mgg31361-bib-0036] Vozarova de Courten, B. , de Courten, M. , Hanson, R. L. , Zahorakova, A. , Egyenes, H. P. , Tataranni, P. A. , … Vozar, J. (2003). Higher prevalence of type 2 diabetes, metabolic syndrome and cardiovascular diseases in gypsies than in non‐gypsies in Slovakia. Diabetes Research and Clinical Practice, 62(2), 95–103. 10.1016/s0168-8227(03)00162-1 14581146

[mgg31361-bib-0037] Walter, S. D. (1989). The feasibility of matching and quota sampling in epidemiologic studies. American Journal of Epidemiology, 130(2), 379–389. 10.1093/oxfordjournals.aje.a115344 2665478

[mgg31361-bib-0038] Werissa, N. A. , Piko, P. , Fiatal, S. , Kosa, Z. , Sandor, J. , & Adany, R. (2019). SNP‐based genetic risk score modeling suggests no increased genetic susceptibility of the Roma population to type 2 diabetes mellitus. Genes, 10(11), 942 10.3390/genes10110942 PMC6896051 31752367

[mgg31361-bib-0039] Xiang, L. , Wu, H. , Pan, A. N. , Patel, B. , Xiang, G. , Qi, L. U. , … Qi, Q. (2016). FTO genotype and weight loss in diet and lifestyle interventions: A systematic review and meta‐analysis. The American Journal of Clinical Nutrition, 103(4), 1162–1170. 10.3945/ajcn.115.123448 26888713 PMC4807705

[mgg31361-bib-0040] Zhao, Y. , Ma, Y. S. , Fang, Y. , Liu, L. , Wu, S. D. , Fu, D. , & Wang, X. F. (2012). IGF2BP2 genetic variation and type 2 diabetes: A global meta‐analysis. DNA and Cell Biology, 31(5), 713–720. 10.1089/dna.2011.1400 22032244

[mgg31361-bib-0041] Zheng, Y. , Ley, S. H. , & Hu, F. B. (2018). Global aetiology and epidemiology of type 2 diabetes mellitus and its complications. Nature Reviews Endocrinology, 14(2), 88–98. 10.1038/nrendo.2017.151 29219149

[mgg31361-bib-0042] Zlatohlavek, L. , Vrablik, M. , Motykova, E. , Ceska, R. , Vasickova, L. , Dlouha, D. , & Hubacek, J. A. (2013). FTO and MC4R gene variants determine BMI changes in children after intensive lifestyle intervention. Clinical Biochemistry, 46(4–5), 313–316. 10.1016/j.clinbiochem.2012.11.017 23201545

